# Olfactory memory networks: from emotional learning to social behaviors

**DOI:** 10.3389/fnbeh.2015.00036

**Published:** 2015-02-17

**Authors:** Regina M. Sullivan, Donald A. Wilson, Nadine Ravel, Anne-Marie Mouly

**Affiliations:** ^1^Emotional Brain Institute, Nathan Kline InstituteOrangeburg, NY, USA; ^2^Child and Adolescent Psychiatry, The Child Study Center, New York University Langone Medical CenterNew York, NY, USA; ^3^Neuroscience and Physiology, Sackler Institute, New York University School of MedicineNew York, NY, USA; ^4^Lyon Neuroscience Research Center, INSERM U1028, CNRS UMR5292, University Lyon1Lyon, France

**Keywords:** olfaction, olfactory memory, social odors, sniffing behavior, odor preference, odor aversion

Odors are powerful stimuli that can evoke emotional states, and support learning and memory. Decades of research have indicated that the neural basis for this strong “odor-emotional memory” connection is due to the uniqueness of the anatomy of the olfactory pathways. Indeed, unlike the other sensory systems, the sense of smell does not pass through the thalamus to be routed to the cortex. Rather, odor information is relayed directly to the limbic system, a brain region typically associated with memory and emotional processes. This provides olfaction with a unique and potent power to influence mood, acquisition of new information, and use of information in many different contexts including social interactions. Indeed, olfaction is crucially involved in behaviors essential for survival of the individual and species, including identification of predators, recognition of individuals for procreation or social hierarchy, location of food, as well as attachment between mating pairs and infant-caretaker dyads. Importantly, odors are sampled through sniffing behavior. This active sensing plays an important role in exploratory behaviors observed in the different contexts mentioned above. Odors are also critical for learning and memory about events and places and constitute efficient retrieval cues for the recall of emotional episodic memories.

This broad role for odors appears highly preserved across species. In addition, the consistent early developmental emergence of olfactory function across diverse species also provides a unique window of opportunity for analysis of myriad behavioral systems from rodents to nonhuman primates and humans. This, when combined with the relatively conserved organization of the olfactory system in mammals, provides a powerful framework to explore how complex behaviors can be modulated by odors to produce adaptive responses, and to investigate the underlying neural networks.

The present research topic brings together cutting edge research on diverse species and developmental stages, highlighting convergence and divergence between humans and animals to facilitate translational research. It is composed of 25 articles and encompasses 5 sections: human olfaction, odor preferences and aversions, odors and social behavior, olfaction and sniffing, and olfactory memory.

## Human odor memory and perception

Pazart et al. (2014) designed an fMRI study to describe the differences in brain activation in wine experts compared to control subjects. They observed specific areas activated in the experts' brains during all phases of wine tasting and reported that wine experts showed a more immediate and targeted sensory reaction to wine stimulation than control subjects. Saive et al. (2014a) reviewed and discussed the different ways of investigating episodic memory in human, ranging from autobiographical approaches to laboratory-based approaches including more ecological paradigms. In an accompanying article, Saive et al. (2014b) presented a novel laboratory-based approach to study episodic memory, which led to the formation and subsequent retrieval of an integrated and multimodal memory of episodes comprising odors (What) localized spatially (Where) within a visual context (Which context). Li (2014) then reviewed rodent and human research in olfactory aversive conditioning, promoting a sensory cortical model of threat perception. By elucidating threat encoding in the sensory cortex, this proposed model may provide new insights into the pathophysiology of emotional disorders, pointing to a concrete clinical intervention target in the sensory cortex. Pool et al. (2014) investigated whether odor discrimination abilities could be enhanced in humans through learning. They used an appetitive Pavlovian conditioning paradigm, during which one of two perceptually non-distinguishable odors was associated with a rewarding taste. They reported a dissociation between explicit perception and physiological reactions: although participants were not able to explicitly perceive a difference, they reacted faster, inhaled more and had higher skin conductance responses when confronted with the reward-associated odor compared to its similar version that was never associated with the reward.

## Odor preferences and aversion

Torquet et al. (2014) designed a new paradigm to study conditioned olfactory preference (odor-sugar) in rats and investigate resulting changes in the hedonic valence of the odors. They showed that discriminative appetitive conditioning induces not only a preference for the reinforced odor, but also a stable devaluation of the non-reinforced stimulus. Aimé et al. (2014) investigated the modulation of olfactory sensitivity and glucose-sensing by the feeding state in obese Zucker rats using a conditioned odor aversion learning paradigm. The data show that obese rats displayed a higher olfactory sensitivity and a 2 fold higher concentration of glucose in the olfactory bulb and cortex than in control lean rats, thus providing strong arguments toward establishing the OB glucose-sensing as a key factor for sensory olfactory processing. Using the same odor aversion paradigm, Ferry (2014) further showed that the orexinergic system which triggers food intake, acts like fasting to increase not only olfactory sensitivity, but also the strength of the odor-malaise association. Finally, Fitzgerald et al. (2014) explored the role of the olfactory tubercle, a structure with known anatomical connectivity with both brain reward and olfactory structures, in regulating odor-motivated behaviors. They confirmed that electrical stimulation of the olfactory tubercle was rewarding and possessed the capacity to alter odor-directed preference behaviors, supporting the notion that the olfactory tubercle is integral to motivated behaviors and likely involved in odor hedonics.

## Odors and social behavior

Brus et al. (2014) investigated the influence of parturition and learning of the lamb odor on neurogenesis in sheep mothers. They reported that learning of the olfactory signature of the lamb, which occurs during the first mother/young interactions, induces a down-regulation in olfactory neurogenesis associated with an enhancement of olfactory neuroblasts maturation. The authors made the assumption that fewer new neurons decrease cell competition in the olfactory bulb and enhance maturation of those new neurons selected to participate in the learning of the young odor. Using another animal model of mother-young interaction in rabbits, Coureaud et al. (2014) addressed the question of memory persistence of odor mixture and components in newborn rabbits. Interacting with the mother during the daily nursing, newborn rabbits experience her body odor cues including the mammary pheromone which promotes the very rapid appetitive learning of simple or complex stimuli (odorants or mixtures) through associative conditioning. The data showed that newborn rabbits have access to both elemental and configural information in certain odor mixtures, and competition between these distinct representations of the mixture influences the persistence of their memories. Social transmission of food preference is another example of social learning involving the olfactory modality. Nicol et al. (2014) showed that this learning can occur under anesthesia and promotes extensive, but spatially distinct, changes in mitral cell networks to both cued and uncued odors as well as in evoked glutamate and GABA release. These data suggest that olfactory learning under anesthesia in mice is supported by broadly similar changes in olfactory bulb encoding as shown previously in conscious paradigms. Another kind of social odor is provided by predator odors. Takahashi (2014) reviewed current research on the olfactory and neural systems that activate fear elicited by predator odors. Interconnections from olfactory systems to brain circuits activated by predator odor are discussed in relation to autonomic, endocrine, and fear-related unconditioned and conditioned fear. Using chronic exposure to a predator or its scent as a social stress, Huo et al. (2014) investigated the role of serotonin as a modulator of the effects of this stress on male-male aggression and sexual attractiveness of urine odor. They found that exposure to a predator or its scent causes down-regulation of male–male aggression and sexual attractiveness of urine odor, and that such inhibitory effects were reduced or abolished in mice lacking brain serotonin. Finally in mice the Grueneberg ganglion located at the tip of the nose close to the entry of the naris, has been identified as a chemodetector of alarm pheromones. Brechbühl et al. (2013) investigated the conserved multisensory modalities of mouse Grueneberg ganglion neurons. They found striking similarities between mouse Grueneberg ganglion neurons and nematode amphid neurons, suggesting that the ability of an organism to detect cues from similar origin occurs in a cluster of specialized olfactory neurons that has been conserved throughout evolution.

## Olfaction and sniffing

Olfaction and respiration are tightly linked since odors are sampled through sniffing behavior. Courtiol et al. (2014) reported that sniffing behavior is not only a matter of odorant sorption properties. Indeed a given odorant was sniffed differently depending on the odor pair in which it was presented. These data suggest that sniffing is adjusted in a synthetic manner that is dependent on the context in which the odorant is presented. Rojas-Líbano et al. (2014) monitored sniffing behavior, simultaneously with the local field potential of the olfactory bulb in rats moving freely in a familiar environment. They found that the local field potential in the olfactory bulb represents the sniff cycle with high reliability at every sniff frequency and can therefore be used to study the neural representation of motor drive in a sensory cortex. Under some circumstances, sniffing behavior can also be used as an index of cognitive processes providing a useful tool at very young ages. Indeed, Boulanger Bertolus et al. (2014) monitored freezing and sniffing behavior during odor fear conditioning at different ages of the rat's life, in order to investigate the learning of interval duration between two events in this paradigm. They observed temporal patterns for freezing and/or respiration curves in pups as young as 12 days post-natal, suggesting that infant rats are able to encode time durations as well as, and as quickly as, adults despite their immature brain. In active rodents, sniffing is often phase locked with other orofacial sensorimotor behaviors like whisking, and head movements. Sirotin et al. (2014) further examined the relationship between sniffing and ultrasonic vocal output of rats in a social environment. They found that ultrasonic vocalization of the 50 kHz family is largely restricted to periods of active sniffing (5–10 Hz), with the calls produced exclusively during exhalations and causing an instantaneous reduction in sniff rate. These results show that ultrasonic vocalizations are an integral part of the rhythmic orofacial behavioral ensemble. In humans, there is psychophysiological evidence that sniffing is modulated by subjective pleasantness of an odor: sniff duration and sniff volume increase when pleasant odors are sampled compared to unpleasant ones. Ferdenzi et al. (2014) investigated how repeated exposure to odors can affect their pleasantness. For this, sniff duration and volume were recorded and paired with ratings of odor pleasantness and intensity. The data showed that affective habituation occurs with repeated exposure, which can be observed both at the self-reported level and on sniffing behavior.

## Olfactory memory

Tong et al. (2014) reviewed some established molecular and structural mechanisms of memory with a focus on the time courses of post-conditioning molecular processes. They described the properties of odor learning intrinsic to the olfactory bulb and reviewed the utility of the olfactory system of adult rodents as a memory system in which to study the cellular mechanisms of cumulative learning. Recent work has begun exploring the role of sleep in olfactory memory. Barnes and Wilson (2014) reviewed recent evidence of sleep-dependent changes in olfactory system structure and function which contribute to odor memory and perception. During slow-wave sleep, the piriform cortex becomes hypo-responsive to odor stimulation and instead displays sharp-wave activity similar to that observed within the hippocampal formation. Furthermore, the functional connectivity between the piriform cortex and other cortical and limbic regions is enhanced during slow-wave sleep compared to waking. This combination of conditions may allow odor memory consolidation to occur during a state of reduced external interference and facilitate association of odor memories with stored hedonic and contextual cues. Olfactory structures have been early reported to exhibit oscillatory population activities which have been proposed to subserve memory processes such as encoding, consolidation and retrieval. Martin and Ravel (2014) reviewed our current knowledge about the conditions in which two main oscillatory rhythms linked to odor processing, namely beta (15–40 Hz) and gamma (60–100 Hz) are observed at the first stages of olfactory processing, the olfactory bulb and the piriform cortex. Based on a critical reexamination of those data, the authors propose hypotheses on the functional involvement of beta and gamma oscillations for odor perception and memory. It is well known from the literature that sensory neural activity is highly context dependent and shaped by experience and expectation. Mandairon et al. (2014) further showed that association of an odorant with a visual context in mice allowed the visual context alone to elicit a behavioral response similar to that elicited by the olfactory stimulus when it was initially presented, and a neural activation pattern in the olfactory bulb that was highly correlated with that elicited by the associated odorant, but not other odorants. The authors concluded that in rodents, neural representation of an odorant in primary sensory areas can be elicited in its absence by exposure to the context to which the odorant was previously associated, further suggesting that rodents can build internal representation of the olfactory stimulus. Finally, Gelperin (2014) underlined that chemosensory processing, and particularly olfactory information processing, is a particularly attractive modality within which to seek comparative insights into cognitive processes underlying learning and memory. He concluded that the study of olfactory information processing may be the most general and fruitful approach to the study of comparative cognition, including consciousness, in the majority of vertebrate animal species.

### Conflict of interest statement

The authors declare that the research was conducted in the absence of any commercial or financial relationships that could be construed as a potential conflict of interest.
